# Revocable Signature Scheme with Implicit and Explicit Certificates

**DOI:** 10.3390/e25091315

**Published:** 2023-09-09

**Authors:** Jerzy Pejaś, Tomasz Hyla, Wojciech Zabierowski

**Affiliations:** 1 Faculty of Computer Science and Information Technology, West Pomeranian University of Technology, 71-210 Szczecin, Poland; jerzy.pejas@zut.edu.pl; 2 Department of Microelectronics and Computer Science, Lodz University of Technology, 93-005 Lodz, Poland

**Keywords:** signature scheme, implicit and explicit certificates-based public key cryptography, bilinear pairing, revocation, non-repudiation

## Abstract

This paper addresses the certificate revocation problem and proposes the first revocable pairing-based signature scheme with implicit and explicit certificates (IE-RCBS-kCAA). We should no longer discuss whether to revoke certificates but how to do it effectively, ensuring both the scalability of the revocation operation and the non-repudiation of the signature in the short or long term. Under the computational difficulty assumptions of the modified collusion attack algorithm with *k* traitors (*k*-mCAA) and discrete logarithm (DL) problems, we demonstrate that our scheme is secure against existential unforgeability under chosen message attacks (EUF-IERCBS-kCAA-CMA) in a random oracle model. The proposed solution is scaled and allows the use of many trusted status authorities that issue explicit short-term certificates confirming the validity of explicit long-term certificates. Furthermore, we demonstrate that our signature scheme has a short-term non-repudiation property for the shell validity model.

## 1. Introduction

Digital signatures are a critical component that ensure the integrity, authenticity and non-repudiation of electronic documents. In public key cryptography (PKC), a digital signature is considered valid if it is mathematically correct and a related certificate is valid. The certificates are issued for a limited period (usually two years), they can be revoked (e.g., a signer’s private key is compromised) and in most cases, a user certificate is issued by an intermediate CA (certificate authority), and other certificates are issued by another intermediate CA or by a root CA with a self-signed certificate. The root CA is considered a trust anchor. All certificates between an end-entity certificate and a trust anchor certificate form the trusted certificate path. The validation of this chain is a challenging task.

Three models for certificate validation exist [1]. In the first one, called the *shell model*, the certificate is valid as long as all the certificates in the certification path are valid when the signature is verified. The second one is the *modified shell model*. In this model, the signing time is the basis for decision making about the signature validity (this requires a timestamp). The third model is the *chain model*: once a signature is valid at signing time, it remains valid all the time. The chain model can only provide non-repudiation of property over a long period of time [1,2].

The purpose of certificate validation is to confirm the authenticity of the public key, i.e., to provide proof that the public key belongs to the signer and that the associated private key was under the sole control of an owner at the moment of signature creation. The process of verifying certificates for signatures that need to be valid for a long period of time is one of the main difficulties when implementing and managing a public key infrastructure. The authenticity of a public key in a public key cryptosystem can be achieved in two ways: either explicitly or implicitly. During explicit authentication, the public key authenticity can be verified directly using the certificate issued by a CA. In implicit authentication, the secret certificate issued by a CA (i.e., partial or complete private key) can be verified indirectly during signature verification or decryption operation. Combining both approaches and obtaining cryptographic schemes based on explicit and implicit certificates is another possible solution (e.g., T. Hyla et al. [3]).

It seemed that the certificate validation problem had been solved in 1984 when Shamir [4] introduced a new identity-based public key cryptography (ID-PKC) concept utilizing a user’s publicly known identity information as her/his public key. Since that time, many schemes based on that concept have been proposed. First came certificateless public key cryptography (CL-PKC), which removed the key escrow problem, one of the major problems in ID-PKC. Next, (implicit) certificate-based public key cryptography (IC-PKC) schemes were proposed. Compared to CL-PKC, IC-PKC schemes are resistant to public key replacement attacks. The certificate is implicit because it must be kept secret and is not used directly during signature verification. In other words, the implicit certificate is a partial secret key sent to the user by a trusted authority (TA) that, in comparison to CL-PKC schemes, additionally binds the user’s identity with its public key and the parameters of the trusted authority. Note that some authors do not use the word “implicit”, which could be misleading as someone might think that certificates are always public.

The great interest shown in the ID-PKC, CL-PKC and IC-PKC schemes is because they eliminate explicit certificates from the encryption or signature schemes and allow the need to manage the status of certificates to be dispensed with. In practice, this is not the case, as the problem of certificate status management is shifted to the user identity management level [5,6]. In addition, this generates problems with public key distribution, such as vulnerability to public key replacement attacks. As a result, key invalidation in ID-PKC, CL-PKC and IC-PKC schemes can be even more cumbersome than in traditional PKC-based cryptosystems.

### 1.1. Related Works

The digital signature schemes must include a revocation mechanism to support non-repudiation and achieve Girault trust level 3 (see Girault, M. [7]). The revocation mechanism in identity-based certificates or based on implicit certificate schemes is implemented through a few techniques: using an online mediator, periodically updating the user’s secret using a secret channel and using time keys that can be sent using a public channel.

In the online mediator (SEM, Security Mediator) technique [8,9], a user’s partial private key is divided into two parts. One part is sent to the user and one to the SEM. The advantage of this approach is instantaneous revocation. The main drawbacks of this approach are the need for a secure (confidential) channel, and that the user cannot create a signature independently. In addition, the SEM must store a large amount of partial secret keys. Secondly, it is possible to generate private keys over regular periods [10,11,12]. When revocation of users is needed, the trusted authority (TA) just stops updating users’ partial private keys. The main drawback is the need for secure channels between users and TA.

In 2001, Boneh and Franklin [13] proposed a method in which a trusted private key generator (PKG) periodically updates private keys for all non-revoked users. Boldyreva et al. [14] introduced the first scalable revocable identity-based encryption scheme. The scheme was later improved by Libert and Vergnaud [15] and by Seo and Emura [16]. In 2012, Tseng and Tsai proposed efficient revocable identity-based encryption [17] and signature schemes [18]. They used a different method of private key construction, where the private key consists of a fixed initial private key and a time key, which is issued periodically by the PKG for non-revoked users. The key can be sent using a public channel. Their work was later reviewed by [19]. Chen et al. [20] proposed a selective-ID secure revocable identity-based encryption (RIBE) scheme from lattices. Cheng et al. [21] presented an adaptive-ID version of the [20] scheme. Lee et al. in [22,23] constructed a RIBE scheme based on pairings using the subset difference method.

In 2014, Sun et al. [24] proposed a revocable certificateless signature (RCLS) scheme in which the TA produces an initial partial private key and a time key corresponding to each period. The time key is transmitted over a public channel. Next, Sun and Shen [25] and Sun et al. [26] proposed a revocable certificateless signature (RCLS) scheme without the use of bilinear pairings.

In 2017, Jia et al. [27] proposed an efficient revocable identity-based signature (RIBS) scheme in which the revocation functionality is outsourced to a cloud revocation server. In their solution, a short-term key (time key) is issued by the cloud server instead of KGC. Recently, a similar solution based on semi-trusted cloud revocation agents (s-CRAs) was used by Ma et al. [28].

### 1.2. Motivation and Contribution

This paper proposes a new revocable signature IE-RCBS-kCAA scheme with implicit and two explicit (short- and long-term) certificates, which is secure in the random oracle model under the hardness assumption of the *modified k-CAA* problem and the discrete logarithm (DL) problem. When an explicit long-term certificate is revoked, its status information (in the form of an explicit short-term certificate) is available online at the moment of signature generation. The method used for revoking and providing certificate status is similar to that presented by Yum et al. [29].

The implicit and short-term explicit certificates are two components of the signature key that can be used to sign documents. A signatory who wishes to reject the signatures he or she has generated may intentionally compromise his or her signature key and falsely claim that the signatures have been forged by someone else. Such a scenario is impossible with an explicit short-term certificate because the signer cannot revoke the explicit short-term certificate used as part of the full signature key.

Using explicit short-term certificates ensures that revocation latency (i.e., a time lag between revoking the certificate and informing the relaying parties) is irrelevant, as the digital signature is valid only until the associated short-term explicit certificate expires. Because of the proposed signature validation approach based on the certificate validation shell model (see Section 1), the acceptance of such a signature should not pose any risk to a relaying party.

**Figure 1:**
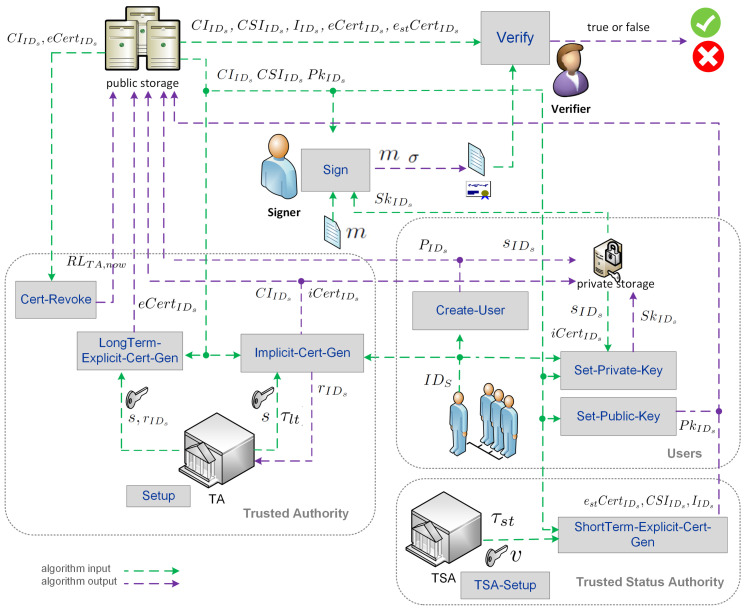
Architecture framework of IE-RCBS-kCAA signature scheme.

In our approach, a user’s private key consists of a secret value, a long-term partial private key and a short-term explicit certificate (a time key). The first two partial keys are kept secret by the user, and their authenticity is confirmed by a long-term certificate issued by the trusted authority (TA). An explicit short-term certificate is periodically updated and sent over a public channel. To perform such an operation (unlike the cloud-based revocation server proposed by Jia et al. in Figure 1), we suggest using the trusted status authority (TSA), which issues a new short-term explicit certificate for each valid long-term explicit certificate and stops doing so when the long-term explicit certificate is revoked.

The IE-RCBS-kCAA scheme fulfils the following conditions:

1. An explicit short-term certificate eliminates the need to generate CRLs used in traditional PKI systems; furthermore, it serves as non-repudiation evidence of a digital signature;
2. During the signature creation process, a three-component user’s private key is used; this approach allows Girault’s trust level 3 security to be achieved; only the verification process, in addition to the public key and explicit signer’s certificates, indirectly references other parties’ keys, including TA keys;
3. A signer’s public key, as in the related two partial private keys, contains three component groups: the signer generates the first, while two others are created by trusted and trusted status authorities;
4. The short- and long-term explicit certificates of a signer are public, i.e., these certificates are used in the signature verification process and to verify their authenticity and their validity;
5. A signature verification process uses short- and long-term explicit certificates, where explicit short-term certificates play a role in the certificate status;
6. The strongest security property for digital signatures is provided, i.e., existential enforceability against adaptively chosen message attacks.

Additionally, in the scheme, a user can only change his public and private keys with TA acceptance and vice versa. A TA cannot generate a false private key of any user to forge a signature without being detected by the user. Hence, the scheme fulfils Girault’s level 3 security requirement.

### 1.3. Paper Organisation

In addition to the introductory section, this paper consists of four sections and two appendices. Section 2 introduces the concept of a signature scheme based on implicit and explicit certificates and its security model against three different attack types. Section 3 proposes a randomized IE-RCBSS-kCAA pairing-based signature scheme, and Section 4 analyses its security in a random oracle. Section 5 presents the analysis of the scheme’s efficiency. The paper ends with conclusions.

## 2. Signature Scheme Framework Architecture and Its Security Model

This section describes the architecture framework of our IE-RCBS-kCAA signature scheme and its security model. The definitions of asymmetric bilinear map groups and the hard computational problems (discrete logarithm (DL) problem, kCAA problem, k-mCAA problem) can be found in Hyla et al. [30] and Mitsunari et al. [31].

### 2.1. Signature Scheme Framework

The architecture framework of the IE-RCBS-kCAA signature scheme is shown in Figure 1. This architecture involves three parties: the trusted authority (TA), the trusted status authority (TSA) and the users (signers and verifiers). At system initialization, the TA generates and publishes common parameters. The TSA can use these parameters to generate its secret master private status key or independently generate its parameters and then use them to generate the secret master private status key. This solution allows the TA and TSA to work in the first case in the same algebraic groups and the second in two different, independent ones.

Next, the TA issues the partial secret key for each registered user using its master system key. The TSA is an authentication service that decides the validity of an explicit long-term certificate at the current time and issues an explicit short-term certificate if the TSA answer is positive. Therefore, on request, the TSA checks the signer’s long-term explicit certificate status according to the signed revocation list from the TA. The revocation list issued at time *t* by a trusted authority, TA, is denoted $RL_{TA,t}$. This list contains the indexes of revoked long-term certificates and is updated periodically. Suppose a signer’s long-term certificate is in the signed revocation list. In that case, the TSA can send back a revoked response with a long-term explicit certificate status value equal to revoked or refuse the request. Otherwise, it outputs good or unknown. This last state indicates that the TSA does not know the certificate being requested.

The trust model (understood as building trust relationships between cryptographic keys and their owner’s identity) with the TSA, and separating its role from the TA role, allows various business models to be obtained that can provide certificate issuing services (explicit or implicit) and determine their status. In a particular case, the TSA may act as an independent third party and provide certificate status verification services to multiple TA authorities. This type of scenario aligns with the model that has long been used in PKI systems: CAs issue certificates, and the OCSP server issues certificates of their status [32].

Let us assume that the earlier mentioned trust authorities and trusted status authorities are part of the trust model based on a common set of system parameters. These parameters may include, among others, the same algebraic groups $G_{1}$, $G_{2}$ and $G_{T}$. It is easy to notice then that such an assumption allows us to effectively solve the scalability problem of the trust architecture shown in Figure 1 and to adapt it to many users. In that case, users can use not only many TSAs but also many TAs. However, this paper only considers the trust model with a single TA and TSA.

The IE-RCBS-kCAA consists of eleven polynomial-time algorithms (compare T. Hyla et al. [33]): the system initialization algorithm (TA-Setup), TSA initialization algorithm (TSA-Setup), secret user’s key generation algorithm (Create-User), implicit certificate extraction algorithm (Implicit-Cert-Gen), long-term explicit certificate generation algorithm (LongTerm-Explicit-Cert-Gen), short-term explicit certificate algorithm (ShortTerm-Explicit-Cert-Gen), full user’s private key creation algorithm (Set-Private-Key), full user’s public key creation algorithm (Set-Private-Key), long-term certificate revocation algorithm (Cert-Revoke) signing algorithm (Sign) and verification algorithm (Verify).

**Definition** **1** (IE-RCBS-kCAA scheme)**.** *An implicit and explicit certificates-based signature against k-traitors collusion attack scheme consists of the following eleven polynomial-time algorithms:*

*TA-Setup $\left(1^{k}\right)\to \left(s,params\right)$. A security parameter $1^{k}$ is an input and outputs the certifier’s master private key s, the system parameters are params and a revocation list $RL_{TA,thisUpdate}$ (initially empty), where thisUpdate indicates the issue date of this RL, which are then properly distributed in the system. The TA runs the algorithm and, when completed, keeps the master private key s secret, while the RL and params are publicly available to the TSA and all other users on the system, respectively.*
*The TA runs the algorithm and, in secret, keeps the master private key s, while the RL and params are publicly accessible to the TSA and all other users in the system, respectively.*
*TSA-Setup $\left(params\right)\to \left(v,V_{0},T_{0}\right)$. The algorithm takes as input the system parameters params and outputs a master private status key v and two related TSA public keys ($V_{0}$, $T_{0}$).*
*Create-User $\left(params,ID_{s}\right)\to \left(s_{ID_{s}},P_{ID_{s}}\right)$. The user runs the algorithm, and the input is the system parameters and the signer’s identity. The output is the user’s secret key value $s_{ID_{s}}$ and the corresponding first partial public key $P_{ID_{s}}$.*
*Implicit-Cert-Gen $\left(params,s,ID_{s},P_{ID_{s}},\tau_{lt}\right)\to \left(CI_{ID_{s}},iCert_{ID_{s}},r_{ID_{s}}\right)$. This algorithm takes as input the system parameter params, master private key s, the identity $ID_{s}$ of a user, its first partial public key $P_{ID_{s}}$ and a certificate validity period $\tau_{lt}$. It outputs the user’s certificate information $CI_{ID_{s}}$, an implicit certificate $iCert_{ID_{s}}$ and the secret key $r_{ID_{s}}$ used by the TA during the user’s implicit and explicit certificates’ generation that is unknown to this user. The TA runs the algorithm once for each user, and the corresponding implicit certificate is distributed to the user secretly.*
*LongTerm-Explicit-Cert-Gen $\left(params,s,CI_{ID_{s}},r_{ID_{s}},q_{ID_{s}}\right)\to \left(eCert_{ID_{s}}\right)$. The input is the system parameter params, master private key s, the user’s certificate information $CI_{ID_{s}}$, the secret key $r_{ID_{s}}$ related to the user’s implicit and explicit certificates, and the hash value $q_{ID_{s}}$. The output is an explicit long-term certificate $eCert_{ID_{s}}$ that is sent to the user by a public channel. A TA runs this algorithm once for each user.*
*ShortTerm-Explicit-Cert-Gen $\left(params,v,V_{0},T_{0},bstr,CI_{ID_{s}},eCert_{ID_{s}},\tau_{st}\right)\to \left(e_{st}Cert_{ID_{s}},CSI_{ID_{s}},I_{ID_{s}}\right)$. This algorithm takes as input the system parameter params, a master private status key v and two related TSA public keys ($V_{0}$, $T_{0}$), the bitstring bstr (e.g., related with the signed message), $CI_{ID_{s}}$ and his/her long-term explicit certificate, and a period $\tau_{st}$. The TSA first checks the current $RL_{TA,t}$. If the request concerns the non-revoked long-term explicit certificate, then the TSA outputs an explicit short-term certificate $e_{st}Cert_{ID_{s}}$, the certificate status information $CSI_{ID_{s}}$ and auxiliary public information $I_{ID_{s}}$ that is sent to the user by a public channel. A TSA runs this algorithm once for each user’s request.*
*Set-Private-Key (params, $CI_{ID_{s}}$, $CSI_{ID_{s}}$, $s_{ID_{s}}$, $iCert_{ID_{s}}$, $e_{st}Cert_{ID_{s}})$→${Sk}_{ID_{s}}$. The user runs this algorithm. The algorithm takes as input the system parameters params, user’s certificate information $CI_{ID_{s}}$, the certificate status information $CSI_{ID_{s}}$, a secret key $s_{ID_{s}}$, an implicit certificate $iCert_{ID_{s}}$ and a short-term explicit certificate $e_{st}Cert_{ID_{s}}$, and returns the corresponding full user’s private key $Sk_{ID_{s}}=\left(s_{ID_{s}},iCert_{ID_{s}},e_{st}Cert_{ID_{s}}\right)$.*
*Set-Public-Key $\left(params,CI_{ID_{s}}\right)\to Pk_{ID_{s}}$: the user S run the algorithm with the certificate information $CI_{ID_{s}}$. It returns the full long-term public key in the form $Pk_{ID_{s}}=\left(P_{ID_{s}},R_{ID_{s}}^{\prime },R_{ID_{s}}^{\prime }\right)$.*
*Cert-Revoke $\left(params,CI_{ID_{s}},eCert_{ID_{s}}\right)\to RL_{TA,thisUpdate}$: for an input tuple $(CI_{ID_{s}}$, $eCert_{ID_{s}})$ with the explicit long-term certificate $eCert_{ID_{s}}$ that is requested to be revoked, the TA verifies entity credentials, and if the entity is authorized successfully, then the TA revokes the certificate and places it on the signed revocation list $RL_{TA,thisUpdate}$ that is issued at thisUpdate.*
*Sign $(params,m,CI_{ID_{s}},Sk_{ID_{s}},Pk_{ID_{s}})\to \sigma$. The signer runs the **Sign** algorithm that generates a signature σ for the given input: the params, a message m, a user certificate information $CI_{ID_{s}}$ and the user’s full key pair $\left(Sk_{ID_{s}},Pk_{ID_{s}}\right)$.*
*Verify (params, (m,σ), $CI_{ID_{s}}$, $CSI_{ID_{s}}$, $I_{ID_{s}}$, $eCert_{ID_{s}}$, $e_{st}Cert_{ID_{s}})$ →{true,false}. Everyone can run the algorithm Verify to check the validity of a signature. Taking as input a message/signature pair (m,σ), a user’s certificate information $CI_{ID_{s}}$, a certificate status information $CSI_{ID_{s}}$, an auxiliary public information $I_{ID_{s}}$, and long- and short-term explicit certificates ($eCert_{ID_{s}}$, $e_{st}Cert_{ID_{s}}$), it outputs true when σ is a valid signature. Otherwise, it outputs false.*

It is required that if σ = Sign (params, *m*, $CI_{ID_{s}}$, $Sk_{ID_{s}}$, $eCert_{ID}$) then Verify (params, *m*, σ, $CI_{ID_{s}}$, $CSI_{ID_{s}}$, $I_{ID_{s}}$, $eCert_{ID_{s}}$, $e_{st}Cert_{ID_{s}}$) = true, where the public parameters params, the signer’s private/public key pair $\left(Sk_{ID_{s}},Pk_{ID_{s}}\right)$ and the long- and short-term explicit certificates ($eCert_{ID_{s}}$, $e_{st}Cert_{ID_{s}}$) are generated based on the specification of the algorithms: TA-Setup, TSA-Setup, Create-User, Implicit-Cert-Gen, LongTerm-Explicit-Cert-Gen and ShortTerm-Explicit-Cert-Gen.

**Remark** **1.** *Implicit-Cert-Gen and Explicit-Cert-Gen algorithms are successful when the TA positively verifies the identity and certificate information of $CI_{ID_{s}}$ confirming this identity. Furthermore, whenever a user requests a certificate for a public key $P_{ID_{s}}$, the user must prove the possession of the corresponding secret key $s_{ID_{s}}$ to the certifier, similar to a traditional public key infrastructure. Similar remarks apply to ShortTerm-Explicit-Cert-Gen: a positive result of this algorithm is only returned for the associated valid long-term unencrypted certificate.*

### 2.2. Security Model

The security proof of the proposed IE-RCBS-kCAA signature scheme is based on the commonly accepted standard security notion EUF-CMA (existential unforgeability under chosen message attack). The EUF-CMA notation guarantees the highest security level of the signature scheme and thus the resistance of the signature scheme against the strongest attacks of the adversary.

The security proofs is a claim made within the random oracle model, where a hash function finally replaces the random oracle. It is easy to see that the last step is heuristic in nature. In practice, the heuristics are successfully used for problem solving (e.g., [34,35,36,37]). However, the security proof in the oracle model can only be treated as a heuristic argument for the security of the cryptographic scheme, but without a guarantee for the security of its real implementation (Bellare and Rogaway [38]).

For the IE-RCBS-kCAA signature scheme, four cases of access or lack of access by an adversary to TA and TSA master keys should be considered:

(a) An adversary does not know the TA and TSA master keys;
(b) An adversary knows the TA and TSA master keys;
(c) An adversary knows the TA master private key and does not know the TSA master private status key;
(d) An adversary does not know the TA master private key TA and knows the TSA master private status key TSA.

Access to or lack of access to TA and TSA keys may depend on the adversary’s knowledge or ignorance of different user keys. Consequently, this allows us to define five different types of adversaries, the capabilities of which are shown in Table 1. Each type of adversary has its role and access rights (yes/no) to the user’s secrets or public key replacement. For example, the $A_{1}$ adversary is a user who has not yet been registered and does not have a certificate. The purpose of the adversary attack is to impersonate this type of user and forge his/her signature. It is assumed that the adversary does not have access to the TA and TSA master keys and to the implicit certificate of the target user but has access to his/her short-term explicit certificate, the secret key, and can change his public key.

Note that even if the $A_{1}$, $A_{3}$ and $A_{4}$ adversaries cannot access the TSA’s master private status key, it still provides them access to the explicit short-term certificate. The TSA is fair and acts as an oracle, responding to any correct requests unless they concern a revoked certificate. In the latter case, the adversary does not receive a valid short-term explicit certificate for the next period. However, acting as a user with the revoked implicit certificate, they can collude with other legal users and generate its correct value.

A thorough analysis of the adversary types and their capabilities in Table 1 shows that the adversaries $A_{1}$ and $A_{5}$ and $A_{2}$ and $A_{4}$ have equivalent capabilities to falsify the target user’s signature. Hence, in the case of the proposed signature scheme with two trust authorities (TA and TSA), only three types of adversaries ($A_{1}$, $A_{2}$ and $A_{3}$), should be considered. As a result, the security model is similar to the models proposed for invalidation signature schemes with a single trust authority (see, e.g., Y. Sun et al. [24] and Y. Huang et al. [39]).

Based on the above comments, the security model of the proposed IE-RCBS-kCAA scheme, from now on referred to as EUF-IERCBS-kCAA-CMA, is defined by three games between challenger *C* and adversary *A*, assuming that the adversary chooses which game to play. In all cases, adversary $A=(A_{1},A_{2},A_{3})$ is trying to break the EUF-CMA security of the IE-RCBS-kCAA scheme, i.e., the formal model describing existential unforgeability. We use two types of adversaries with different capabilities: Type I adversary and Type II adversary (see, e.g., [3]) to describe the first two games. For the third type of adversary, i.e., Type III adversary, we adopt the security notation introduced by Y. Sun et al. [24] and Y. Huang et al. [39] that is necessary for the security proofs to come. Type I and II adversaries are similar to those defined in [30] and their descriptions are omitted here. Type III adversary ($A_{3}$) represents a revoked certified user whose long-term explicit certificate is no longer valid. However, it should be noted that a revoked user still holds her/his implicit certificate and related secret key. However, the TSA stops issuing the subsequent short-term explicit certificates to her/him. The adversary cannot gain the TA’s master secret keys and the TSA’s master private status key but can replace the public key of any user, except the target user, with a value of her/his choice. The security model categorises potential adversaries based on their attack capabilities and classifies Type I/II/III adversaries into three categories (see Li, J., et al. [40,41,42] and Huang, X., et al. [43]): Normal adversary, Strong adversary and Super adversary. The scheme should resist a Super Type I/II/III adversary (in Games I/II/III), who can obtain a valid signature under the public key chosen by itself without providing the corresponding secret.

**Definition** **2.** *An implicit and explicit certificate revocable signature scheme IE-RCBS-kCAA has existential unforgeability against chosen message attacks (EUF-IERCBS-kCAA-CMA) if no probabilistic polynomial-time adversary has a non-negligible probability of winning Game I, Game II and Game III.*

## 3. A Novel Revocable Implicit and Explicit Certificates-Based Signature Scheme

### 3.1. The Revocable Signature Scheme with Common System Parameters (IE-RCBS-kCAA)

The IE-RCBS-kCAA scheme consists of eleven polynomial-time algorithms: TA-Setup, TSA-Setup, Create-User, Implicit-Cert-Gen, LongTerm-Explicit-Cert-Gen, Set-Private-Key, Cert-Revoke, Get-Cert-Status, Sign and Verify. The algorithms are as follows.

1. **TA-Setup**: The system parameters are $params=\{G_{1}$, $G_{2}$, $G_{T}$, *p*, $\hat{e}$, *P*, $P_{0}$, *Q*, $Q_{0}$, $H_{1}$, $H_{2}$, $H_{3}\}$, where $|G_{1}\left|=\right|G_{2}\left|=\right|G_{T}|=p$ for some prime number $p\geq 2^{k}$ (*k* is the system security number), (P,Q) are generators of, respectively, $G_{1}$ and $G_{2}$ such that $\hat{e}\left(P,Q\right)=g$, $P_{0}=sP$ and $Q_{0}=sQ$, the system’s master public keys with the master private key $s\in Z_{p}^{*}$, $H_{1},H_{2}:\Gamma \to Z_{p}$ and $H_{3}:{\left\{0,1\right\}}^{*}\to Z_{p}$ are three secure cryptographic hash functions. Γ means a string space that defines a user with the identity ID. When ID contains more information other than the identity, we mark it as CI or CSI.
2. **TSA-Setup** (params): The TSA chooses a random number $v\in Z_{p}^{*}$ as its master private status key and calculates its public keys $V_{0}=vP$ and $T_{0}=vQ$.
3. **Create-User** ($params,ID_{s}$): The user $ID_{s}$ chooses a random number $s_{ID_{s}}\in Z_{p}^{*}$, sets $s_{ID_{s}}$ as the secret key and produces the corresponding first partial long-term public key $P_{ID_{s}}=s_{ID_{s}}P$. The secret key $s_{ID_{s}}$ is kept secret, while the user sends $P_{ID_{s}}$ to the TA over an authenticated channel.
4. **Implicit-Cert-Gen** ($params,s,ID_{s},P_{ID_{s}},\tau_{lt}$): Given $ID_{s}$ presenting S’s identity, his partial long-term public key $P_{ID_{s}}$ and a period $\tau_{lt}$, the trust authority TA:
  (a) Randomly selects $r_{ID_{s}}\in Z_{p}^{*}$ and computes respective second and third partial long-term public keys $\left(R_{ID_{s}}^{\prime },R_{ID_{s}}^{\prime \prime }\right)=(r_{ID_{s}}P$, $r_{ID_{s}}Q)$;
  (b) Composes the user’s certificate information $CI_{ID_{s}}$, including the TA’s public keys $\left(P_{0},Q_{0}\right)$, identifiers $ID_{s}$ and $ID_{TA}$ of the user S and the TA, respectively, first, second and third partial public keys ($P_{ID_{s}},R_{ID_{s}}^{\prime },R_{ID_{s}}^{\prime \prime }$), and the period $\tau_{lt}$ for which the information $CI_{ID_{s}}$ is valid;
  (c) For $P_{ID_{s}}$ and $\left(R_{ID_{s}}^{\prime },R_{ID_{s}}^{\prime \prime }\right)$ computes:
    (1) $$q_{ID_{s}}=H_{1}\left(CI_{ID_{s}}\right)$$
  (d) Generates S’s partial private key (an implicit certificate):
    (2) $$iCert_{ID_{s}}=\frac{1}{s+r_{ID_{s}}q_{ID_{s}}}Q$$
    and transmits it to the user S secretly; in addition, TA sends $CI_{ID_{s}}$.
5. **LongTerm-Explicit-Cert-Gen** $\left(params,s,CI_{ID_{s}},r_{ID_{s}},q_{ID_{s}}\right)$: The TA generates the signer’s S explicit certificate using parameters provided by S and the values created when executing the **Implicit-Cert-Gen** algorithm:
  (a) The TA creates the explicit certificate that links S’s identity with the public key components:
    (3) $$eCert_{ID_{s}}=\frac{1}{s+r_{ID_{s}}q_{ID_{s}}}P$$
  (b) The TA sends $eCert_{ID_{s}}$ to an entity S.
6. **ShortTerm-Explicit-Cert-Gen** ($params,v,V_{0},T_{0},bstr,CI_{ID_{s}},eCert_{ID_{s}},\tau_{st}$): Taking as input any bitstring, the user’s certificate information $CI_{ID_{s}}$ and his/her long-term explicit certificate $eCert_{ID_{s}}$ (created for the period $\tau_{lt}$) and a period $\tau_{st}$, the TSA first checks if the user and his/her long-term explicit certificate are in the $RL_{TA,t}$. If that is so, the TSA rejects the update request. Otherwise, the TSA:
  (a) Randomly selects secret key $z\in Z_{p}^{*}$ and computes $\left(Z^{\prime },Z^{\prime \prime }\right)=(zP$, zQ);
  (b) Composes the certificate status information $CSI_{ID_{s}}$, including $\left(Z^{\prime },Z^{\prime \prime }\right)$, the TSA public keys $\left(V_{0},T_{0}\right)$, $ID_{s}$ and $ID_{TSA}$ identifiers, the status value equal to good, and the period $\tau_{st}$ for which the information $CSI_{ID_{s}}$ should be valid;
  (c) For $CI_{ID_{s}}$, an explicit certificate $eCert_{ID_{s}}$ and $CSI_{ID_{s}}$ computes:
    (4) $$\begin{array}{cc} t_{ID_{s}}= & H_{2}\left(bstr,CI_{ID_{s}},eCert_{ID_{s}},CSI_{ID_{s}}\right)\\ I_{ID_{s}}= & \left(v+zt_{ID_{s}}\right)\left(Q_{0}+q_{ID_{s}}R_{ID_{s}}^{\prime \prime }\right) \end{array}$$
    where $q_{ID_{s}}=H_{1}\left(CI_{ID_{s}}\right)$;
  (d) Generates the explicit short-term certificate (the certificate status evidence) as:
    (5) $$e_{st}Cert_{ID_{s}}=\frac{1}{v+zt_{ID_{s}}}Q$$
    and transfers it to the user *S* via a public (open) channel; in addition, the TSA sends $CSI_{ID_{s}}$ and $I_{ID_{s}}$.
7. **Set-Private-Key** $\left(params,CI_{ID_{s}},CSI_{ID_{s}},s_{ID_{s}},iCert_{ID_{s}},e_{st}Cert_{ID_{s}}\right)$: The user S calculates the hash values $q_{ID_{s}}$ and $t_{ID_{s}}$ (see Equations (1) and (4)), and checks if $\hat{e}\left(q_{ID_{s}}R_{ID_{s}}^{\prime }+P_{0},iCert_{ID_{s}}\right)=\hat{e}\left(t_{ID_{s}}Z_{ID_{s}}^{\prime }+V_{0},e_{st}Cert_{ID_{s}}\right)=\hat{e}\left(P,Q\right)=g$; if in both cases the answer is positive, then the algorithm formulates a full private key in the form $Sk_{ID_{s}}=\left(s_{ID_{s}},iCert_{ID_{s}},e_{st}Cert_{ID_{s}}\right)$.
8. **Set-Public-Key** $\left(params,CI_{ID_{s}}\right)$: The user S with $P_{ID_{s}}$, $R_{ID_{s}}^{\prime }$ and $R_{ID_{s}}^{\prime \prime }$ (taken from the user’s certificate information $CI_{ID_{s}}$) sets his full long-term public key in the form $Pk_{ID_{s}}=\left(P_{ID_{s}},R_{ID_{s}}^{\prime },R_{ID_{s}}^{\prime \prime }\right)$. The TA publishes the resulting full long-term public key in its public repository and distributes it to all interested parties.
9. **Cert-Revoke** ($params,CI_{ID_{s}},eCert_{ID_{s}})$: The user with $CI_{ID_{s}}$ or any other authorized entity sends to TA a tuple $(CI_{ID_{s}}$, $eCert_{ID_{s}})$ with the explicit long-term certificate $eCert_{ID_{s}}$ to be revoked. After verifying the entity credentials to revoke the certificate, TA revokes it and places it on a signed revocation list $RL_{TA,t}$.
10. **Sign** $\left(params,m,CI_{ID_{s}},Sk_{ID_{s}},eCert_{ID_{s}}\right)$: To sign a message $m\in {\left\{0,1\right\}}^{*}$, a signer *S* performs the following steps:
  (a) Picks two random numbers $k_{1},k_{2}\in_{R}Z_{p}^{*}$;
  (b) Computes the hash value $bstr=H_{3}(m,k_{1}P$), and $q_{ID_{s}}=H_{1}\left(CI_{ID_{s}}\right)$;
  (c) Generates a short-term explicit certificate by calling the ShortTerm-Explicit-Cert-Gen ($params,v,V_{0},T_{0},bstr,CI_{ID_{s}},eCert_{ID_{s}},\tau_{st}$) $\to (e_{st}Cert_{ID_{s}},CSI_{ID_{s}},I_{ID_{s}})$ function;
  (d) Generates the signature $\sigma =(h,w_{1},w_{2},E)$,
    (6) $$E=\frac{k_{1}-k_{2}^{-1}h}{k_{1}h+s_{ID_{s}}}\left(iCert_{ID_{s}}+e_{st}Cert_{ID_{s}}\right)$$
    where $h=H_{3}\left(m,k_{1}P,U,q_{ID_{s}}\right)$, $w_{1}=k_{1}-hs_{ID_{s}}\;\left(\mathrm{mod}\;p\right)$, $w_{2}=k_{2}\left(k_{1}h+s_{ID_{s}}\right)\;\left(\mathrm{mod}\;p\right)$, while $U=e{\left(P,T_{0}+t_{ID_{s}}Z{\prime \prime }_{ID_{s}}+Q_{0}+q_{ID_{s}}R{\prime \prime }_{ID_{s}}\right)}^{k_{1}k_{2}}$;
  (e) If in (6) $k_{1}h+s_{ID_{s}}=0$, then repeat steps (a) and (b).
  **Note**. Each time a signature is generated, a fresh short-term explicit certificate is retrieved from the TSA (cf. ShortTerm-Explicit-Cert-Gen algorithm).
11. **Verify** $\left(params,m,\sigma ,CI_{ID_{s}},CSI_{ID_{s}},I_{ID_{s}},eCert_{ID_{s}},e_{st}Cert_{ID_{s}}\right)$: To verify the tuple containing the message, the signature and certificates, i.e., $(m,\sigma =\left(h,w_{1},w_{2},E\right)$, $eCert_{ID_{s}}$, $e_{st}Cert_{ID_{s}}$, $I_{ID_{s}})$, V performs the following steps:
  (a) Computes $q_{ID_{s}}$ (see Equation (1)) and then calculates values:
    (7) $$\begin{array}{cc} U^{\prime }= & \hat{e}{\left(\psi (I_{ID_{s}}),E\right)}^{w_{2}}\hat{e}{\left(eCert_{ID_{s}}+\psi \left(e_{st}Cert_{ID_{s}}\right),I_{ID_{s}}\right)}^{h}\\ \bar{k_{1}P}= & w_{1}P+hP_{ID_{s}} \end{array}$$
  (b) Computes $bstr=H_{3}(m,k_{1}P$) and $t_{ID_{s}}$ (see Equation (4));
  (c) If the status of the certificate $eCert_{ID_{s}}$ in the certificate status information $CSI_{ID_{s}}$ is correct and (8) is valid, then returns *accept*, otherwise *reject*.
    (8) $$h\equiv H_{3}\left(m,\bar{k_{1}P},U^{\prime },q_{ID_{s}}\right)$$

**Remark** **2.** *Note that during the indirect signature verification, the long- and short-term explicit certificates are validated ($eCert_{ID}$ and $etCert_{ID}$, respectively). This verification can also be performed directly based on the following formulas:*

(9) $$\begin{array}{c} g\equiv \hat{e}\left(eCert_{ID_{s}},q_{ID_{s}}R{\prime \prime }_{ID_{s}}+Q_{0}\right)\\ g\equiv \hat{e}\left(t_{ID_{s}}Z_{ID_{s}}^{\prime }+V_{0},e_{st}Cert_{ID_{s}}\right) \end{array}$$

*If the conditions formulated in Equations (8)–(9) are met, it means that a signature is mathematically correct. It is the first postulate for a digital signature to be valid. The second one applies to the validity of digital signatures at a semantical level that depends on the underlying validity model (Baier, H. et al. [1]).*

*Suppose we use a shell model and the verifier received the signature at time $t_{v}$ called the verification time. Assuming that the TA’s master private key and the TSA’s master private status key are irrevocable signature keys, the semantic validity of the digital signature depends on a short- and long-term certificate validity ($e_{st}Cert$ and eCert, respectively). Because both certificates are mathematically correct and ($e_{st}Cert$, eCert) certificates are issued with respective periods $\tau_{lt}=\left[\tau_{lt}^{i},\tau_{lt}^{e}\right]$, $\tau_{st}=\left[\tau_{st}^{i},\tau_{st}^{e}\right]$ and expiry dates $\tau_{lt}^{e}$, $\tau_{st}^{e}$, then a verifier checks if:*

*(a)*  *$e_{st}Cert$ was certified by the TSA and the validity period $\tau_{st}$ of $e_{st}Cert$ satisfies $\tau_{lt}^{i}\leq \tau_{st}^{i}<\tau_{st}^{e}\leq \tau_{lt}^{e}$;*
*(b)*  *$t_{v}\in \left[\tau_{st}^{i},\tau_{st}^{e}\right]$.*

*When the above conditions are successful, the signature will be accepted as valid short-period non-repudiation evidence in whole period $\tau_{st}=\left[\tau_{st}^{i},\tau_{st}^{e}\right]$.*

**Remark** **3.** *Based on the properties of the asymmetric bilinear map groups:*

(10) $$eCert_{ID_{s}}=\psi \left(iCert_{ID_{s}}\right)$$

*Hence, it follows that, alternatively, the execution of the **LongTerm-Explicit-Cert-Gen** algorithm can be entrusted to the signatory S, who, after receiving the implicit certificate from the TA will use Equation (10) to calculate the explicit certificate.*

### 3.2. Correctness

The $\sigma =(h,w_{1},w_{2},E)$ is a valid signature on message *m* because it is accepted by **Verify**. We state the proof as follows:

(11) $$\begin{array}{cc} U^{\prime }= & \hat{e}{\left(\psi (I_{ID_{s}}),E\right)}^{w_{2}}\hat{e}{\left(eCert_{ID_{s}}+\psi \left(e_{st}Cert_{ID_{s}}\right),I_{ID_{s}})\right)}^{h}\\ = & \hat{e}{\left(\psi \left(I_{ID_{s}}\right)),\frac{k_{1}-k_{2}^{-1}h}{k_{1}h+s_{ID_{s}}}\left(iCert_{ID_{s}}+e_{st}Cert_{ID_{s}}\right)\right)}^{k_{2}\left(k_{1}h+s_{ID_{s}}\right)}\\ \; & \hat{e}{\left(eCert_{ID_{s}}+\psi \left(e_{st}Cert_{ID_{s}}\right),I_{ID_{s}}\right)}^{h}\\ = & \hat{e}\left(\psi \left(I_{ID_{s}}\right),\left(k_{1}k_{2}-h\right)\left(iCert_{ID_{s}}+e_{st}Cert_{ID_{s}}\right)\right)\\ \; & \hat{e}\left(\psi \left(I_{ID_{s}}\right),h\left(iCert_{ID_{s}}+e_{st}Cert_{ID_{s}}\right)\right)\\ = & \hat{e}\left(\psi \left(\left(v+zt_{ID_{s}}\right)\left(Q_{0}+q_{ID_{s}}R_{ID_{s}}^{\prime \prime }\right)\right),k_{1}k_{2}\left(\frac{1}{s+r_{ID_{s}}q_{ID_{s}}}+\frac{1}{v+zt_{ID_{s}}}\right)Q\right)\\ = & \hat{e}{\left(P,\left(s+q_{ID_{s}}r_{ID_{s}}\right)Q+\left(v+zt_{ID_{s}}\right)Q\right)}^{k_{1}k_{2}}\\ = & \hat{e}{\left(P,Q_{0}+q_{ID_{s}}R{\prime \prime }_{ID_{s}}+T_{0}+t_{ID_{s}}Z{\prime \prime }_{ID_{s}}\right)}^{k_{1}k_{2}}=U \end{array}$$

Thus,

(12) $$\begin{array}{c} h^{\prime }=H_{3}\left(m,\bar{k_{1}P},U^{\prime },q_{ID_{s}}\right)=H_{3}\left(m,w_{1}P+hP_{ID_{s}},U^{\prime },q_{ID_{s}}\right)=h \end{array}$$

Moreover, based on this, it is straightforward to prove the correctness of the long-term explicit certificate:

(13) $$\begin{array}{cc} g^{\prime } & =\hat{e}\left(eCert_{ID_{s}},q_{ID_{s}}R{\prime \prime }_{ID_{s}}+Q_{0}\right)=\hat{e}\left(\frac{1}{s+r_{ID_{s}}q_{ID_{s}}}P,\left(q_{ID_{s}}r_{ID_{s}}+s\right)Q\right)\\ \; & =\hat{e}\left(P,Q\right)=g \end{array}$$

and short-term explicit certificate:

(14) $$\begin{array}{cc} g^{\prime } & =\hat{e}\left(t_{ID_{s}}Z_{ID_{s}}^{\prime }+V_{0},e_{st}Cert_{ID_{s}},\right)=\hat{e}\left(\left(t_{ID_{s}}z+v\right)P,\frac{1}{v+zt_{ID_{s}}}Q,\right)\\ \; & =\hat{e}\left(P,Q\right)=g \end{array}$$

## 4. Security Analysis

In Games I and II, the TSA is treated as an oracle that answers every query the challenger or adversary asks. It has been assumed that long-term certificates are not revoked in these two games. Therefore, all explicit short-term certificates issued by the TSA have the status *good*. In Game III, long-term certificates can be revoked. The TSA will not issue a short-term explicit certificate for the next validity period of $\tau_{st_{i}}$. Because the adversary still owns the implicit and long-term explicit certificates, it can try to produce valid signatures even if the previous short-term explicit certificate is no longer valid. The adversary does not know its short explicit certificate for the new target period but can cooperate with legal users to obtain such a certificate.

We proved the IE-RCBS-kCAA scheme security by reducing the security of a higher-level construction to a lower-level primitive. In particular, we reduced the existence of an adversary by transforming the protocol into an algorithm that solves the corresponding k-mCAA problem or the discrete logarithm (DL) problem with non-negligible probability. To this end, we used a general forking lemma (Bellare and Neven [44]), similar to [30].

Table 1 below shows that in comparison with the $A_{1}$ and $A_{3}$ adversaries, the $A_{2}$ adversary’s capabilities are greater (if only because he/she has access to the master private key and master private status key that belong to the TA and TSA, respectively). On the other hand, the capabilities of $A_{1}$ and $A_{3}$ adversaries are similar:

- $A_{3}$ knows the implicit certificates of users whose long-term explicit certificate has been revoked (in particular, it may be his/her certificate) but cannot obtain from the TSA any valid short-term explicit certificates related to them; the TSA will not respond to any request of the adversary to issue an explicit short-term certificate for the next period after the related long-term explicit certificate has been revoked; hence, the adversary, in order to forge the adversary’s signature, must be able to calculate an explicit short-term certificate;
- $A_{1}$ does not know the implicit certificates of users who were indicated as targets of the adversary attack; however, since, in this case, none of the explicit long-term certificates were revoked, the TSA responds to every request to issue (also from the adversary) a short-term explicit certificate for the next validity period; hence, the adversary $A_{1}$ knows the explicit short-term certificates of all users, including those who are the targets of the attack, but must calculate the corresponding implicit certificates.

In both cases, after creating a valid forged signature, the adversaries $A_{1}$ and $A_{3}$ disclose the corresponding short- and long-term explicit certificate. It follows that challenger *C* with the help of adversary $A_{1}$ or $A_{3}$ could solve the computing k-mCAA problem. However, this is contrary to the assumption that the k-mCAA problem is a computationally difficult problem. Hence, the proposed IE-RCBS-kCAA signature scheme is provably secure against Types I and III adversaries, as demonstrated in Lemmas 1 and 3, respectively. In Lemma 2, we also prove that IE-RCBS-kCAA is secure against a Type II adversary.

**Lemma** **1.** *Suppose the hash functions $H_{1}$, $H_{2}$ and $H_{3}$ are random oracles, and $A_{1}$ is a Type I adversary in Game I against the IE-RCBS-kCAA scheme. When the adversary $A_{1}$ has a non-negligible ϵ advantage over the IE-RCBS-kCAA scheme, then there is a reduction $R_{1}$ that solves the k-mCAA problem over the $G_{2}$ group with non-negligible probability:*

(15) $$\varepsilon_{k-mCAA}^{R_{1}}\geq \frac{\varepsilon^{2}}{\gamma^{2}e{\left(\left(q_{I}+q_{E}+q_{S}\right)+1\right)}^{2}}$$

*where e is the base of the natural logarithm, $q_{I}$, $q_{E}$, c$q_{S}$ and $\gamma =q_{H_{3}}$ are the upper bound on the number of queries sent to the respective Implicit-Cert-Gen-Query, LongTerm-Explicit-Cert-Gen-Query, Super-Sign-Query oracles and the $H_{3}$-Query oracle.*

**Proof.**  (sketch) According to the approach given in [30] (also compare Lemma 3), our reduction consists of two phases. First, we apply the intermediate algorithm $B_{1}$ (i.e., the wrapper) that interacts with adversary $A_{1}$ and it returns a side output. Second, we build a reduction algorithm $R_{1}$ that launches general forking algorithm $F_{B_{1}}$ with wrapper $B_{1}$ that handles the simulation of the IE-RCBS-kCAA scheme environment to the actual adversary. The algorithm $R_{1}$ returns data that allow the correct solution of the *k*-mCAA problem to be obtained.

Assume that $B_{1}$ is given a random instance $▵=(G_{2},p,P,sP,Q$, sQ, ${\left(s+r_{1}q_{1}\right)}^{-1}Q$, …, ${\left(s+r_{k}q_{k}\right)}^{-1}Q)$ of the *k*-mCAA problem, where $G_{2}$ is a group with a large prime order p. For the master private key $s\in Z_{p}^{*}$ unknown to *C* and $B_{1}$, the goal is to compute ${\left(r^{*}q^{*}+s\right)}^{-1}Q$ for some $q^{*}\notin \left\{q_{1},\ldots ,q_{k}\right\}$, $r^{*}Q\notin$ $\{r_{1}Q$, …, $r_{k}Q\}$, and given $q_{1},\ldots ,q_{k}\in Z_{p}^{*}$, $r_{1}Q$, …, $r_{k}Q$. In order to achieve this goal, we convert Type I adversary $A_{1}$ to algorithm $B_{1}$ (compare with Lemma 3). Finally, the reduction algorithm $R_{1}$ invokes a general forking algorithm $F_{B_{1}}$ with the wrapper $B_{1}$ to solve the challenge ▵.

Note that in comparison to Lemma 3, the simulation of *ShortTerm-Explicit-Cert-Gen-Query* is simpler because it is reasonable now to respond to each request of the adversary $A_{1}$ (no long-term certificate is revoked). What is more, this response is always provided by the TSA, which thus becomes a component of the simulation environment.

$R_{1}$ obtains two signature forgeries $\hat{\sigma_{i}}=$ $(\hat{m}$, ${\hat{h}}_{i},\hat{w_{1,i}},\hat{w_{2,i}},{\hat{E}}_{i}$, $P_{ID}$, $eCert_{ID}$, $e_{st}Cert_{ID}$, $I_{ID})$, (i=0,1) for the message $\hat{m}$, partial public key $P_{ID}$, and long- and short-term explicit certificates $eCert_{ID}$, $e_{st}Cert_{ID}$. If both forgeries are valid, then $R_{1}$ obtains two sets of side outputs $\sigma_{0}$ and $\sigma_{1}$ where $\sigma_{i}$ (for i=0,1) is written as $({\hat{\beta }}_{i},{\hat{h}}_{i},\hat{t_{i}},\hat{w_{2,i}},\hat{c_{i}},{\hat{U}}_{i},{\hat{E}}_{i},P_{ID}$, ${\hat{C}}_{ID}$, ${\hat{CSI}}_{ID}$, ${\hat{R}}_{ID}^{\prime }$, ${\hat{R}}_{ID}^{\prime \prime }$, *C*, $eCert_{ID}$, $e_{st}Cert_{ID}$, $I_{ID})$. Moreover, we assume that ${\hat{U}}_{0}={\hat{U}}_{1}$, $q^{*}={\hat{c}}_{0}={\hat{c}}_{1}$ and $R_{ID}^{\prime \prime }=r^{*}Q$ (compare Lemma 3). $R_{1}$ outputs *failure* and stops if both ${\hat{\beta }}_{0}$ and ${\hat{\beta }}_{1}$ are equal to 0.

Based on $\sigma_{0}$ and $\sigma_{1}$, the following equation is used:

(16) $$\begin{array}{c} \hat{e}{\left(\psi \left(I_{ID}\right),{\hat{E}}_{0}\right)}^{{\hat{w}}_{2,0}}\hat{e}{\left(eCert_{ID}+\psi \left(e_{st}Cert_{ID}\right),I_{ID}\right)}^{{\hat{h}}_{0}}=\\ =\hat{e}{\left(\psi \left(I_{ID}\right),{\hat{E}}_{1}\right)}^{{\hat{w}}_{2,1}}\hat{e}{\left(eCert_{ID}+\psi \left(e_{st}Cert_{ID}\right),I_{ID}\right)}^{{\hat{h}}_{1}} \end{array}$$

Equation (16) can be converted into:

(17) $$\begin{array}{c} \hat{e}\left(\psi \left(I_{ID}\right),{\hat{w}}_{2,0}{\hat{E}}_{0}+{\hat{h}}_{0}\left(iCert_{ID}+e_{st}Cert_{ID}\right)\right)=\\ =\hat{e}\left(\psi \left(I_{ID}\right),{\hat{w}}_{2,1}{\hat{E}}_{1}+{\hat{h}}_{1}\left(iCert_{ID}+e_{st}Cert_{ID}\right)\right) \end{array}$$

Eventually, the solution to the *k*-mCAA problem is:

(18) $$iCert_{ID_{s}}=\frac{1}{\left(r^{*}q^{*}+s\right)}Q=\frac{\left({\hat{w}}_{2,0}{\hat{E}}_{0}-{\hat{w}}_{2,1}{\hat{E}}_{1}\right)}{\left({\hat{h}}_{0}-{\hat{h}}_{1}\right)}-e_{st}Cert_{ID}$$

where $q^{*}\notin \left\{q_{1},\ldots ,g_{k}\right\}$ and $r^{*}Q\notin \left\{r_{1}Q,\ldots ,r_{k}Q\right\}$. Note that a public channel transmits all short-term explicit certificates. Hence, in particular, $e_{st}Cert_{ID_{s}}$ can be known both to adversary $A_{1}$ and algorithm $R_{1}$.

The success probability of a Super Type I adversary is calculated similarly to Lemma 3. We should consider the same four events $\neg E_{1}$, $\neg E_{2}$, $\neg E_{3}$ and $\neg E_{1}$ with one exception: the wrapper $B_{1}$ cannot fail during the simulation of the oracle *ShortTerm-Explicit-Cert-Gen-Query*. Finally, from the general forking lemma, the success probability $\varepsilon_{k-mCAA}^{R_{1}}$ can be expressed as in Equation (15).

*This ends the sketch proof.*    □

Next, in Game II, applied to the Super Type II adversary where the adversary models the certified entity, we require that signers are honest and the TA registers their tuples $\left(ID,P_{ID},eCert_{ID}\right)$. The following lemma can be shown for this assumption with the use of a random oracle model:

**Lemma** **2.** *Suppose the hash functions $H_{1}$, $H_{2}$ and $H_{3}$ are random oracles, and $A_{2}$ is a Type II adversary in Game II against the IE-RCBS-kCAA scheme. When the adversary $A_{2}$ has a non-negligible ϵ advantage over the IE-RCBS-kCAA scheme, there is a reduction $R_{2}$ that solves the DL problem over the $G_{1}$ group with non-negligible probability:*

(19) $$\varepsilon_{k-mCAA}^{R_{2}}\geq \frac{\varepsilon^{2}}{\gamma e{\left(\left(q_{R}+q_{C}\right)+1\right)}^{2}}$$

*where $q_{R}$, $q_{C}$ and $\gamma =q_{H_{2}}$ are the upper bound on the number of respective queries sent to the Public-Key-Replacement-Query, Corruption-Query and $H_{2}$-Query oracles.*

The proof is similar to the proof of [30] and is omitted here.

**Lemma** **3.** *Suppose the hash functions $H_{1}$, $H_{2}$ and $H_{3}$ are random oracles, and $A_{3}$ is a Type III adversary in Game III against the IE-RCBS-kCAA scheme. When the adversary $A_{3}$ has a non-negligible ϵ advantage over the IE-RCBS-kCAA scheme, there is a reduction $R_{3}$ that solves the k-mCAA problem over the $G_{2}$ group with non-negligible probability:*

(20) $$\varepsilon_{k-mCAA}^{R_{3}}\geq \frac{\varepsilon^{2}}{\gamma e{\left(\left(q_{I}+q_{E}+q_{T}+q_{S}\right)+1\right)}^{2}}$$

*where $q_{I}$, $q_{E}$, $q_{T}$, $q_{S}$ and $\gamma =q_{H_{3}}$ are the upper bound on the number of respective queries sent to the Implicit-Cert-Gen-Query, LongTerm-Explicit-Cert-Gen-Query, ShortTerm-Explicit-Cert-Gen-Query, Super-Sign-Query and $H_{3}$-Query oracles.*

**Proof.**  We begin by describing the $B_{3}$ wrapper and next demonstrate how $R_{3}$ reduction invokes the $F_{B_{3}}$ algorithm on the $B_{3}$ wrapper to solve the *k*-mCAA problem. Suppose the adversary $A_{3}$ can make $q_{H_{1}}$, $q_{H_{2}}$, $q_{H_{3}}$, $q_{T}$ and $q_{S}$ queries to hash functions $H_{1}$, $H_{2}$, $H_{3}$, and the *ShortTerm-Explicit-Cert-Gen-Query* and *Super-Sign-Query* oracle.

Algorithm $R_{3}$ is given a random instance $▵=(G_{1},G_{2},p,P,sP,Q$, sQ, ${\left(s+r_{1}q_{1}\right)}^{-1}Q$, …, ${\left(s+r_{k}q_{k}\right)}^{-1}Q)$ of the *k*-mCAA problem, where the master private key $s\in Z_{p}^{*}$ is unknown to *C* and $B_{3}$. The challenger *C* and direct algorithm $R_{3}$ are asked to calculate ${\left(r^{*}q^{*}+s\right)}^{-1}Q$ for some $q^{*}\notin \left\{q_{1},\ldots ,q_{k}\right\}$, $r^{*}Q\notin$ $\{r_{1}Q$, …, $r_{k}Q\}$, and given $q_{1},\ldots ,q_{k}\in Z_{p}^{*}$, $r_{1}Q$, …, $r_{k}Q$.

Assume we are also given $t_{1},\ldots ,t_{k}\in Z_{p}^{*}$, $z_{1}Q$, …, $z_{k}Q$, $z_{1}P$, …, $z_{k}P$, ${\left(v+z_{1}t_{1}\right)}^{-1}Q$, …, ${\left(v+z_{k}t_{k}\right)}^{-1}Q)$, $\left(v+z_{1}t_{1}\right)\left(sQ+q_{1}r_{1}Q\right)$, …, $\left(v+z_{k}t_{k}\right)\left(sQ+q_{k}r_{k}Q\right)$. This allows us to simulate the *ShortTerm-Explicit-Cert-Gen-Query* behaviour for all unrevoked long-term implicit certificates.

1. The Wrapper

We demonstrate that Type III adversary $A_{3}$ can be converted to algorithm $B_{3}$ and then used to solve a random instance ▵ of the *k*-mCAA problem. Assume that $\gamma =q_{H_{3}}$ and $H=Z_{p}$. Wrapper $B_{3}$ takes ▵ as an argument with a set of random elements $q_{1},\ldots ,q_{k}\in Z_{p}^{*}$ and $h_{1},\ldots ,h_{\gamma }\in Z_{p}^{*}$ and returns a tuple (J,σ) where *J* refers to indices of the target $H_{3}$ query and where σ is the side output. $B_{3}$ maintains two counters ctr and cin, which are initially both set to one, and three lists $L_{H_{1}}$, $L_{H_{2}}$ and $L_{H_{3}}$ used to store the answers to the $H_{1}$, $H_{2}$ and $H_{3}$ random oracle queries. Wrapper $B_{3}$ interacts with adversary $A_{3}$ as follows (Algorithm 1).

**Algorithm 1** *B_3_*(▵).*Initialize*. ctr=1, cin=1, lists $L_{H_{1}}$, $L_{H_{2}}$ and $L_{H_{3}}$ are empty.
*TA-Setup*. $B_{3}$ sets *P* and *Q* as the generators of groups $G_{1}$ and $G_{2}$, respectively, sets TA’s master public keys ($P_{0}=sP$, $Q_{0}=sQ$) and TSA’s master public status keys ($V_{0}=vP$, $T_{0}=vQ$). We assume that master secret keys *s* and *v* are unknown to everyone, including $B_{3}$. Then, $B_{3}$ defines $params=\{G_{1}$, $G_{2}$, $G_{T}$, *p*, $\hat{e}$, $P,P_{0},V_{0},Q,Q_{0}$, $T_{0}$, $H_{1}$, $H_{2}$, $H_{3}\}$ and sends them to the adversary $A_{3}$.
**Queries**: $A_{3}$ can query the following oracles polynomial number of times.

*Create-User-Query* (params, ID). Let us assume that the query is about the identity of ID and that $B_{3}$ replies as described below:
(a)$B_{3}$ scans list $L_{U}$ with tuples in form $\langle ID_{i},s_{ID_{i}},Pk_{ID_{i}}\rangle$ to check whether $ID_{i}=ID$ and if it is true returns a previously defined value $P_{ID_{i}}$.(b)else, $B_{3}$ selects $s_{ID}\in_{R}Z_{p}$ at random and calculates public key $P_{ID}=s_{ID}P$. $B_{1}$ returns $P_{ID}$ and stores the tuple $\langle ID_{i},s_{ID},P_{ID}\rangle$ in the $L_{U}$ list.
*$H_{1}$-Query*$\left(CI_{ID_{i}}\right)$. Algorithm $B_{3}$ maintains a list $L_{H_{1}}$ of tuples $\langle CI_{ID_{i}},P_{ID_{i}}$, $R_{ID_{i}}^{\prime }$, $R_{ID_{i}}^{{}^{\prime \prime }}$, $coin_{i}$, $cin_{i}$, $c_{i},C_{i},eCert_{ID_{i}}\rangle$. If $B_{3}$ or $A_{3}$ queries $H_{1}$, algorithm $B_{3}$ returns $c_{i}$ directly when $L_{H_{1}}$ contains a tuple $\langle CI_{ID_{i}}$, $P_{ID_{i}}$, $R_{ID_{i}}^{\prime }$, $R_{ID_{i}}^{{}^{\prime \prime }}$, $coin_{i}$, $cin_{i}$, $c_{i},C_{i},eCert_{ID_{i}}\rangle$. Else:
(a)$B_{3}$ randomly selects $c\in_{R}Z_{p}^{*}$ and sets $coin=C=eCert_{ID_{i}}=⊥$ when the query is made explicitly by $A_{3}$ (⊥ denotes unknown fields to $B_{3}$);(b)else, $B_{3}$ flips a biased coin that outputs value coin=1 with a probability of ς and coin=0 with a probability of 1−ς (the ς will be optimized later); next:
i.if coin=0, $B_{3}$ selects $cin^{th}$ (1≤cin≤k) value $q_{cin}\in \left\{q_{1},\ldots ,q_{k}\right\}$ and sets $c=q_{cin}$, $C={\left(r_{cin}c+s\right)}^{-1}Q$, $eCert_{ID_{i}}=\psi \left(C\right)$, $R_{ID_{i}}^{\prime \prime }=r_{cin}Q$ and $R_{ID_{i}}^{\prime }=\psi \left(R_{ID_{i}}^{{}^{\prime \prime }}\right)$;ii.else, if $coin_{i}=1$, $B_{3}$ randomly selects $c\in_{R}Z_{p}^{*}$ and $R_{ID}^{{}^{\prime \prime }}\in G_{2}$ such that $c\notin \{q_{1},\ldots ,q_{k}\}$ and $R_{ID}^{{}^{\prime \prime }}\notin$ $\{r_{1}Q$, …, $r_{k}Q\}$, respectively; computes $R_{ID}^{\prime }=\psi \left(R_{ID}^{{}^{\prime \prime }}\right)$ and sets *C* = $eCert_{ID_{i}}$ = ⊥;
(c)$\langle CI_{ID_{i}},P_{ID_{i}}$, $R_{ID_{i}}^{\prime }$, $R_{ID_{i}}^{{}^{\prime \prime }}$, coin, cin, *c*, *C*, $eCert_{ID_{i}}\rangle$ is stored in $L_{H_{1}}$ and returns *c* as the answer.
*$H_{2}$-Query*$(CI_{ID_{i}}$, $eCert_{ID_{i}}$, $CSI_{ID_{s}})$. On receiving the $H_{2}$ query on $(CI_{ID_{i}}$, $eCert_{ID_{i}}$, $CSI_{ID_{s}})$, algorithm $B_{3}$ looks up the list $L_{H_{2}}$. If the corresponding entry already appears in $L_{H_{2}}$ with a tuple $\langle CI_{ID_{i}}$, $eCert_{ID_{i}}$, $P_{ID_{i}}$, $CSI_{ID_{s}}$, $Z_{ID_{i}}^{\prime }$, $Z_{ID_{i}}^{{}^{\prime \prime }}$, $cin_{i}$, $coin_{i}$, $t_{i}$, $e_{st}Cert_{ID_{i}}$, $I_{ID_{i}}\rangle$, then $B_{3}$ responds with $t_{i}$. Otherwise:
(a)if the query is made explicitly by $A_{3}$, $B_{3}$ randomly selects $t\in_{R}Z_{p}^{*}$ and sets coin=1, $e_{st}Cert_{ID_{i}}=I_{ID_{i}}=⊥$;(b)otherwise, $B_{3}$ first call *$H_{1}$-Query*$\left(CI_{ID_{i}}\right)$ oracle and as a result takes a tuple $\langle CI_{ID_{i}}$, $P_{ID_{i}}$, $R_{ID_{i}}^{\prime }$, $R_{ID_{i}}^{{}^{\prime \prime }}$, $coin_{i}$, $cin_{i}$, $c_{i},C_{i},eCert_{ID_{i}}\rangle$ form $L_{H_{1}}$ list.
i.if $coin_{i}=0$, $B_{3}$ chooses $t_{cin_{i}}\in \left\{t_{1},\ldots ,t_{k}\right\}$ and sets $t=t_{cin_{i}}$, $e_{st}Cert_{ID_{i}}$ = ${\left(v+z_{cin}t\right)}^{-1}Q$, $Z_{ID_{i}}^{\prime }=z_{cin}P$, $Z_{ID_{i}}^{{}^{\prime \prime }}=z_{cin}Q$, $I_{ID_{i}}$=$\left(v+z_{cin}t\right)\left(sQ+c_{i}R_{ID_{i}}^{{}^{\prime \prime }}\right)$ii.otherwise, if $coin_{i}=1$, $B_{3}$ randomly selects $t\in_{R}Z_{p}^{*}$ and $Z_{ID}^{{}^{\prime \prime }}\in G_{2}$ such that $t\notin \{t_{1},\ldots ,t_{k}\}$ and $Z_{ID}^{{}^{\prime \prime }}\notin$ $\{z_{1}Q$, …, $z_{k}Q\}$, respectively; computes $Z_{ID}^{\prime }=\psi \left(Z_{ID}^{{}^{\prime \prime }}\right)$ and sets $e_{st}Cert_{ID_{i}}=I_{ID_{i}}=⊥$;
(c)$\langle CI_{ID_{i}}$, $eCert_{ID_{i}}$, $P_{ID_{i}}$, $CSI_{ID_{s}}$, $Z_{ID_{i}}^{\prime }$, $Z_{ID_{i}}^{{}^{\prime \prime }}$, $cin_{i}$, $coin_{i}$, *t*, $e_{st}Cert_{ID_{i}}$, $I_{ID_{i}}\rangle$ is stored in $L_{H_{2}}$ and *t* is output as the answer.
*$H_{3}$-Query*(m, $k_{1}P$, *U*, $H_{1}\left(CI_{ID_{i}}\right))$. Algorithm $B_{3}$ maintains a list $L_{H_{3}}$ of tuples $\langle m_{i}$, ${\left(k_{1}P\right)}_{i}$, $U_{i},c_{i},ctr,w_{2,i},h_{i}\rangle$, where $c_{i}$=$H_{1}\left(CI_{ID_{i}}\right)$. $B_{3}$ runs *$H_{1}$-Query*$\left(CI_{ID_{i}}\right)$ and gets requested hash value *c*. For each request made on $\left(m,k_{1}P,U,c\right)$, algorithm $B_{3}$ returns $h_{i}$ directly when $L_{H_{3}}$ contains tuple $\langle m_{i},{\left(k_{1}P\right)}_{i},U_{i},c_{i},ctr$, $w_{2,i},h_{i}\rangle$. Else, $B_{3}$ returns $h=h_{ctr}\in_{R}Z_{p}^{*}$ as the output, adds tuple $\langle m_{i},k_{1}P,U$, *c*, ctr,⊥,h〉 to $L_{H_{3}}$ and increments ctr by one.*Public-Key-Replacement-Query*$\left(ID,P_{ID},P_{ID}^{\prime }\right)$:
(a)$B_{3}$ tries to find a tuple $\langle ID_{i},s_{ID_{i}},P_{ID_{i}}\rangle$ in the $L_{U}$ list such that $ID_{i}=ID$ and $P_{ID_{i}}=P_{ID}$. When this does not exist, $B_{3}$ outputs ⊥.(b)Else, $B_{3}$ replaces $\langle ID_{i},s_{ID_{i}},P_{ID_{i}}\rangle$ with $\langle ID_{i},⊥,P_{ID_{i}}^{\prime }\rangle$. In this case, the secret value associated with the new public key is not necessary to replace the public key.*Corruption-Query (ID)*. $B_{3}$ browses the list $L_{U}$ for ID and tries to find a tuple $\langle ID_{i},s_{ID_{i}}$, $P_{ID_{i}}\rangle$, then returns $s_{ID_{si}}$ to $A_{3}$ when the user ID is registered. If this is not the case, $B_{3}$ selects a random number $s_{ID}\in_{R}Z_{p}$, sets $P_{ID}=s_{ID}P$, adds $\langle ID,s_{ID},P_{ID}\rangle$ to the $L_{U}$ list and returns $s_{ID_{i}}$ to $A_{3}$.*Implicit-Cert-Gen-Query* ($ID,P_{ID}$). At any moment, $A_{3}$ or $B_{3}$ can query this oracle based on identity ID and partial public key $P_{ID}$.
(a)On the running *Implicit-Cert-Gen-Query* for ID and $P_{ID}$, $B_{3}$ first checks list $L_{U}$. When a user with ID is not created, $B_{3}$ returns ⊥.(b)Now, $B_{3}$ tries to find tuple $\langle CI_{ID_{i}},P_{ID_{i}}$, $R_{ID_{i}}^{\prime }$, $R_{ID_{i}}^{{}^{\prime \prime }}$, $coin_{i}$, $cin_{i}$, $c_{i}$, $C_{i}$, $eCert_{ID_{i}}\rangle$ in $L_{H_{1}}$ that fulfils the following conditions: $CI_{ID_{i}}.ID\equiv ID$, $P_{ID_{i}}\equiv P_{ID}$ and $coin_{i}\neq ⊥$. If such a tuple exists, then:
i.if $coin_{i}=1$, *failure* (denoted by $E_{11}$) is returned and the simulation stops because it cannot respond to a query about any revoked user with an identity of ID and partial public key $P_{ID}$;ii.otherwise, $B_{3}$ outputs $(C_{i},R_{ID_{i}}^{\prime }$, $R_{ID_{i}}^{{}^{\prime \prime }},CI_{ID_{i}})$ to $A_{3}$ as the answer.
(c)Otherwise, $B_{3}$
i.creates $CI_{ID}$ (see *Implicit-Cert-Gen* algorithm in Section 3.1), where $R_{ID}^{\prime }$ = $R_{ID}^{{}^{\prime \prime }}$ = ⊥;ii.runs *$H_{1}$-Query*$\left(CI_{ID}\right)$ and repeats step (b).

*LongTerm-Explicit-Cert-Gen-Query* ($CI_{ID},P_{ID}$). Upon receiving a query on identity ID with certificate information $CI_{ID}$ and partial public key $P_{ID}$:
(a)$B_{3}$ first checks list $L_{U}$. If a user with $CI_{ID}.ID$ is not created, $B_{1}$ returns ⊥.(b)If there is a tuple $\langle CI_{ID_{i}},P_{ID_{i}}$, $R_{ID_{i}}^{\prime }$, $R_{ID_{i}}^{{}^{\prime \prime }}$, coin, $cin_{i}$, $c_{i}$, $C_{i}$, $eCert_{ID_{i}}\rangle$ in $L_{H_{1}}$ such that $CI_{ID_{i}}\equiv CI_{ID}$, $P_{ID_{i}}\equiv P_{ID}$ and $coin_{i}\neq ⊥$, then:
i.if $coin_{i}=1$, *failure* (denoted by $E_{12}$) is output and the simulation stops because it is not allowed to answer the query on any revoked user with the certificate information $CI_{ID}$, which is to be challenged;ii.otherwise, it outputs $(eCert_{ID_{i}},R_{ID_{i}}^{\prime }$, $R_{ID_{i}}^{\prime \prime },CI_{ID_{i}})$ to $A_{3}$ as the answer.
(c)Otherwise, $B_{3}$ runs *$H_{1}$-Query*$\left(CI_{ID}\right)$ and repeats step (b).
*ShortTerm-Explicit-Cert-Gen-Query* ($CI_{ID},P_{ID}$, $eCert_{ID}$).
(a)$B_{1}$ verifies list $L_{U}$. When a user with $CI_{ID}.ID$ does not exists, $B_{1}$ returns ⊥.(b)Now, $B_{3}$ checks in $L_{H_{2}}$ list if there is a tuple $\langle CI_{ID_{i}}$, $eCert_{ID_{i}}$, $P_{ID_{i}}$, $CSI_{ID_{s}}$, $Z_{ID_{i}}^{\prime }$, $Z_{ID_{i}}^{\prime \prime }$, $cin_{i}$, $coin_{i}$, *t*, $e_{st}Cert_{ID_{i}}$, $I_{ID_{i}}\rangle$ such that $CI_{ID}\equiv CI_{ID_{i}}$, $P_{ID}\equiv P_{ID_{i}}$, $eCert_{ID}\equiv eCert_{ID_{i}}$ and $coin_{i}\neq ⊥$. Provided that such a tuple exists, then:
i.if $coin_{i}=1$, *failure* (denoted by $E_{13}$) is returned the simulation stops since $B_{1}$ is not allowed to respond to the query on any revoked user with certificate information $CI_{ID}$, which is to be challenged;ii.otherwise, $B_{3}$ outputs $(e_{st}Cert_{i}$, $CSI_{ID_{i}}$, $I_{ID_{i}})$;
(c)Otherwise, $B_{3}$
i.composes a certificate status information $CSI_{ID}$ (see *ShortTerm-Explicit-Cert-Gen* algorithm in Section 3.1), where $Z_{ID}^{\prime }$ = $Z_{ID}^{\prime \prime }$ = ⊥;ii.runs *$H_{2}$-Query*$(CI_{ID}$, $eCert_{ID}$, $CSI_{ID_{s}})$ and repeats step (b).

*Super-Sign-Query* ($m,CI_{ID},CSI_{ID},P_{ID}$). $B_{3}$ returns ⊥ if a user with CI.ID does not exists.
Else:
(a)if there is no a tuple $\langle CI_{ID_{i}},P_{ID_{i}}$, $R_{ID_{i}}^{\prime }$, $R_{ID_{i}}^{\prime \prime }$, $coin_{i}$, $cin_{i}$, $c_{i}$, $C_{i}$, $eCert_{ID_{i}}\rangle$ in $L_{H_{1}}$ that fulfils the following conditions: $CI_{ID_{i}}\equiv CI_{ID}$, $P_{ID_{i}}\equiv P_{ID}$ and $coin_{i}\neq ⊥$, $B_{3}$ runs *$H_{1}$-Query*$\left(CI_{ID}\right)$;(b)if tuple $\langle CI_{ID_{i}}$, $eCert_{ID_{i}}$, $P_{ID_{i}}$, $CSI_{ID_{s}}$, $Z_{ID_{i}}^{\prime }$, $Z_{ID_{i}}^{\prime \prime }$, $cin_{i}$, $coin_{i}$, *t*, $e_{st}Cert_{ID_{i}}$, $I_{ID_{i}}\rangle$ does not exists in $L_{H_{2}}$ such that $CI_{ID}\equiv CI_{ID_{i}}$, $P_{ID}\equiv P_{ID_{i}}$, $CSI_{ID}\equiv CSI_{ID_{i}}$ and $coin_{i}\neq ⊥$, $B_{3}$ runs *$H_{2}$-Query*$(CI_{ID}$, $eCert_{ID}$, $CSI_{ID_{s}})$;(c)next, if $coin_{i}=1$, $B_{3}$ reports *failure* (denoted by $E_{14}$) and terminates the simulation because it is not allowed to answer the sign query on any revoked and challenged user with certificate information $CI_{ID}$;(d)otherwise $B_{3}$ calculates the signature as follows:
i.sets $c=c_{i}$, $eCert_{ID}=eCert_{ID_{i}}$, $e_{st}Cert_{ID}=e_{st}Cert_{ID_{i}}$, $I_{ID}$ = $I_{ID_{i}}$;ii.selects $w_{1},w_{2}\in_{R}Z_{p}^{*}$ and $E\in_{R}G_{1}$ at random and sets $h=h_{ctr}$;iii.calculates $U=\hat{e}{\left(\psi (I_{ID}),E\right)}^{w_{2}}\hat{e}{\left(eCert_{ID}+\psi \left(e_{st}Cert_{ID}\right),I_{ID})\right)}^{h}$ and $k_{1}P=w_{1}P+hP_{ID}$;iv.$B_{3}$ tries to find tuple $\left(m,k_{1}P,U,c\right)$ in the $L_{H_{3}}$ list; if such a tuple appears in tuple $\langle m,{\left(k_{1}P\right)}_{i}$, $U_{i},c_{i},ctr_{i},w_{w,i},h_{i}\rangle$ of the $L_{H_{2}}$ list, i.e., $m=m_{i}$, $k_{1}P={\left(k_{1}P\right)}_{i}$, $U=U_{i}$ and $c=c_{i}$, $B_{3}$ increment index ctr by one and repeats from step (b) and point (ii);v.$B_{3}$ adds tuple $\langle m,k_{1}P$, $U,c,ctr,w_{2},h\rangle$ to $L_{H_{3}}$ and increments ctr by one;vi.$B_{3}$ returns tuple $(m,\sigma =\left(h,w_{1},w_{2},E\right)$, $CI_{ID}$, $CSI_{ID}$, $eCert_{ID}$, $e_{st}Cert_{ID}$, $I_{ID})$ to $A_{3}$, where σ is the signature.


          The sign query oracle does not use the user’s secret value, which makes it a *Super-Sign* oracle.          *Output*. A successful adversary returns a valid forgery $(\hat{m},\hat{\sigma }=\left(\hat{h},\hat{w_{1}},\hat{w_{2}},\hat{E}\right)$, $CI_{ID},CSI_{ID},eCert_{ID},e_{st}Cert_{ID},I_{ID})$ for $(\hat{m}$, $CI_{ID}$, $CSI_{ID}$, $P_{ID})$. Hence, we have $\hat{h}=$ $H_{3}\left(\hat{m},\hat{w_{1}}P+\hat{h}P_{ID},U,\hat{c}\right)$, where *U* = $\hat{e}(\psi \left(I_{ID}\right)$, $\hat{E}{)}^{{\hat{w}}_{2}}$ $\hat{e}(eCert_{ID}$+$\psi (e_{st}Cert_{ID})$, $I_{ID}{)}^{\hat{h}}$, $\hat{c}=H_{1}\left(CI_{ID}\right)$ and $P_{ID}={\hat{s}}_{ID}P$. In this instance, $P_{ID}$ is chosen by $A_{3}$ and may not be the one returned by the oracle *Create-User-Query*. Moreover, $(CI_{ID},P_{ID}$, $eCert_{ID})$ and $(\hat{m},CI_{ID},CSI_{ID},$ $P_{ID})$ have never appeared as *ShortTerm-Explicit-Cert-Gen-Query* or *Super-Sign-Query* queries, respectively.          Let $\langle CI_{ID},P_{ID}$, $R_{ID}^{\prime }$, $R_{ID}^{\prime \prime }$, coin, cin, $\hat{c}$, *C*, $eCert_{ID}\rangle$, $\langle CI_{ID}$, $eCert_{ID}$, $P_{ID}$, $CSI_{ID}$, $Z_{ID}^{\prime }$, $Z_{ID}^{\prime \prime }$, cin, coin, $\hat{t}$, ⊥, ⊥〉 and $\langle \hat{m}$, $\hat{w_{1}}P+\hat{h}P_{ID},U,\hat{c},ctr_{i}$, $\hat{w_{2}}$, $\hat{h}\rangle$ be the respective tuples of $L_{H_{1}}$, $L_{H_{2}}$ and $L_{H_{3}}$ that correspond to the target valid forgery $\hat{\sigma }$. Thus, wrapper $B_{3}$ returns $(ctr_{i}$, coin, $\hat{h}$, $\hat{t}$, $\hat{w_{2}}$, $\hat{c}$, *U*, $\hat{E}$, $P_{ID}$, ${\hat{CI}}_{ID}$, ${\hat{CSI}}_{ID}$, ${\hat{R}}_{ID}^{\prime }$, ${\hat{R}}_{ID}^{\prime \prime }$, *C*, $eCert_{ID}$, $e_{st}Cert_{ID}$, $I_{ID})$ as its output.          Note that side output σ consists of $(coin,\hat{h},\hat{t},\hat{w_{2}}$, $\hat{c}$, *U*, $\hat{E}$, $P_{ID}$, ${\hat{CI}}_{ID}$, ${\hat{CSI}}_{ID}$, ${\hat{R}}_{ID}^{\prime }$, ${\hat{R}}_{ID}^{\prime \prime }$, *C*, $eCert_{ID}$, $e_{st}Cert_{ID}$, $I_{ID})$. In order to achieve these side output components, we assume that tuple $\left(\hat{m},\hat{w_{1}}P+\hat{h}P_{ID},U,\hat{c}\right)$ has been queried to random oracle *$H_{3}$-Query* and the tuple $\langle \hat{m}$, $\hat{w_{1}}P+\hat{h}P_{ID},U,\hat{c},ctr_{i},\hat{w_{2}},\hat{h}\rangle$ is given in $L_{H_{3}}$ list).          When an adversary returns an *invalid* forgery, $B_{3}$ returns *failure* (denoted by $E_{2}$) and aborts.

2. Reduction Algorithm $R_{3}$

Now we can show how to build a reduction algorithm $R_{3}$ that can exploit the general forking algorithm related with the above wrapper $B_{3}$. Let $▵=(G_{1},G_{2},p,P,sP,Q$, sQ, ${\left(s+r_{1}q_{1}\right)}^{-1}Q$, …, ${\left(s+r_{k}q_{k}\right)}^{-1}Q)$ be the given *k*-mCAA problem. Reducing Algorithm $R_{3}$ invokes the general forking algorithm $F_{B_{3}}$ to solve the *k*-mCAA problem (Algorithm 2).

**Algorithm 2** *R_3_*(▵).$\left(b,\left\{\sigma_{0},\sigma_{1}\right\}\right)\underset{\leftarrow }{\$}F_{B_{3}}\left(▵\right)$ if (b == 0) then return 0 // Event E3 ($F_{B_{3}}$ fails and stops) parse $\sigma_{i}$ as $({\hat{\beta }}_{i},\hat{h_{i}},\hat{t_{i}},\hat{w_{2,i}}$, $\hat{c_{i}}$, ${\hat{U}}_{i}$, $\hat{E_{i}}$, $P_{ID}$, ${\hat{CI}}_{ID}$,${\hat{CSI}}_{ID}$,                  ${\hat{R}}_{ID}^{\prime }$, ${\hat{R}}_{ID}^{\prime \prime }$, *C*, $eCert_{ID}$, $e_{st}Cert_{ID}$, $I_{ID})$ let $\hat{\beta }={\hat{\beta }}_{0}={\hat{\beta }}_{1},{\hat{U}}_{0}={\hat{U}}_{1}$, $q^{*}$=${\hat{c}}_{0}={\hat{c}}_{1}$ and ${\hat{t}}_{0}={\hat{t}}_{1}$ if $\hat{\beta }==1$ then  
      return $iCert_{ID}={\left({\hat{h}}_{0}-{\hat{h}}_{1}\right)}^{-1}\left(\hat{w_{2,0}}{\hat{E}}_{0}-\hat{w_{2,1}}{\hat{E}}_{1}\right)-e_{st}Cert_{ID}$ else return 0 // Event E4 ($F_{B_{3}}$
is successful)

3. Correctness of the k-mCAA Problem Solution

When the general forking algorithm $F_{B_{3}}$ does not fail, $R_{3}$ obtains two sets of side outputs $\sigma_{0}$ and $\sigma_{1}$, where $\sigma_{i}$ (for i=0,1) is written as $({\hat{\beta }}_{i},\hat{h_{i}},\hat{t_{i}},\hat{w_{2,i}}$, $\hat{c_{i}}$, ${\hat{U}}_{i}$, $\hat{E_{i}}$, $P_{ID}$, ${\hat{C}}_{ID}$, ${\hat{CSI}}_{ID}$, ${\hat{R}}_{ID}^{\prime }$, ${\hat{R}}_{ID}^{\prime \prime }$, *C*, $eCert_{ID}$, $e_{st}Cert_{ID}$, $I_{ID})$. Compare output σ from wrapper $B_{3}$ to measure this phenomenon. Additionally, we assume that $\hat{\beta }={\hat{\beta }}_{0}={\hat{\beta }}_{1},{\hat{U}}_{0}={\hat{U}}_{1}$, $q^{*}={\hat{c}}_{0}={\hat{c}}_{1}$, ${\hat{t}}_{0}={\hat{t}}_{1}$ and $R_{ID}^{\prime \prime }=r^{*}Q$. $R_{3}$ returns *failure* (denoted by $E_{4}$) and stops if $\hat{\beta }$ is equal to 0.

Algorithm $R_{3}$ obtains two valid signature forgeries $\hat{\sigma_{i}}$ = $(\hat{m}$, $\hat{\sigma }=(\hat{h_{i}}$, $\hat{w_{1,i}}$, $\hat{w_{2,i}}$, $\hat{E_{i}}$), CI, CSI, eCert, $e_{st}Cert_{ID}$, $I_{ID})$ (i=0,1) for the same message $\hat{m}$, public key $P_{ID}$, long-term explicit certificate $eCert_{ID}$ and short-term explicit certificate $e_{st}Cert_{ID}$. The following equation is applied based on two sets of side outputs $\sigma_{0}$ and $\sigma_{1}$:

(21) $$\begin{array}{c} \begin{array}{c} \hat{e}{\left(\psi \left(I_{ID}\right),{\hat{E}}_{0}\right)}^{{\hat{w}}_{2,0}}\hat{e}{\left(eCert_{ID}+\psi \left(e_{st}Cert_{ID}\right),I_{ID}\right)}^{{\hat{h}}_{0}}=\\ =\hat{e}{\left(\psi \left(I_{ID}\right),{\hat{E}}_{1}\right)}^{{\hat{w}}_{2,1}}\hat{e}{\left(eCert_{ID}+\psi \left(e_{st}Cert_{ID}\right),I_{ID}\right)}^{{\hat{h}}_{1}} \end{array} \end{array}$$

By making suitable arrangements:

(22) $$\begin{array}{c} \begin{array}{c} \hat{e}\left(\psi \left(I_{ID}\right),{\hat{w}}_{2,0}{\hat{E}}_{0}+{\hat{h}}_{0}\left(iCert_{ID}+e_{st}Cert_{ID}\right)\right)=\\ =\hat{e}\left(\psi \left(I_{ID}\right),{\hat{w}}_{2,1}{\hat{E}}_{1}+{\hat{h}}_{1}\left(iCert_{ID}+e_{st}Cert_{ID}\right)\right) \end{array} \end{array}$$

Eventually, the *k*-mCAA problem solution is:

(23) $$iCert_{ID}=\frac{1}{\left(r^{*}q^{*}+s\right)}Q=\frac{\left({\hat{w}}_{2,0}{\hat{E}}_{0}-{\hat{w}}_{2,1}{\hat{E}}_{1}\right)}{\left({\hat{h}}_{0}-{\hat{h}}_{1}\right)}-e_{st}Cert_{ID}$$

where $q^{*}\notin \left\{q_{1},\ldots ,q_{k}\right\}$ and $\hat{r^{*}}Q\notin \left\{r_{1}Q,\ldots ,r_{k}Q\right\}$.

The probability that the $R_{3}$ algorithm will solve the *k*-mCAA problem has not yet been calculated. In accordance with the simulation results, the $R_{3}$ algorithm can compute the value of $iCert_{ID}$ if and only if the below events occur:

$\neg E_{1}:$$B_{3}$ does not fail during the simulation;
$\neg E_{2}:$$A_{3}$ outputs a valid forgery;
$\neg E_{3}:$$F_{B_{3}}$ does not fail;
$\neg E_{4}:$$R_{3}$ does not fail, i.e., in interaction with adversary $A_{3}$ outputs two valid forgeries with a coin value $\hat{\beta }$ of 1.

We denote the probability with which $F_{B_{3}}$ succeeds during the first run as $acc_{3}$. Since $F_{B_{3}}$ succeeds during the first run when there is no interruption in the query phase (event $E_{1}$ does not occur) and when adversary $A_{3}$ creates a valid forgery (event $E_{2}$ does not occur), we have:

(24) $$acc_{3}\geq Pr\left[\neg E_{1}\wedge \neg E_{2}\right]=Pr\left[\neg E_{1}\right]Pr\left[\neg E_{2}|\neg E_{1}\right]$$

Event $\neg E_{1}$ occurs only when the four following events for $\neg E_{1}$ happen:

$\neg E_{11}:$$B_{3}$ cannot terminate during oracle simulation *Implicit-Cert-Gen-Query*, which occurs with a probability of ${\left(1-\zeta \right)}^{q_{I}}$;
$\neg E_{12}:$$B_{3}$ cannot terminate during oracle simulation *LongTerm-Explicit-Cert-Gen-Query*, which occurs with a probability of ${\left(1-\zeta \right)}^{q_{E}}$;
$\neg E_{13}:$$B_{3}$ cannot terminate during oracle simulation *ShortTerm-Explicit-Cert-Gen-Query*, which occurs with a probability of ${\left(1-\zeta \right)}^{q_{T}}$;
$\neg E_{14}:$$B_{3}$ cannot terminate during oracle simulation *Super-Sign-Query*, which occurs with a probability of ${\left(1-\zeta \right)}^{q_{S}}$.

Then we obtain:

(25) $$Pr\left[\neg E_{1}\right]=Pr\left[\neg E_{11}\wedge \neg E_{12}\wedge \neg E_{13}\wedge \neg E_{14}\right]={\left(1-\zeta \right)}^{\mu }$$

where $\mu =q_{I}+q_{E}+q_{T}+q_{S}$.

In addition, the probability of adversary $A_{3}$ producing a valid forgery when event $E_{1}$ does not occur is equal to $Pr[\neg E_{2}|\neg E_{1}]=\varepsilon$.

If events $E_{3}$ and $E_{4}$ do not occur, then the advantage of the algorithm $R_{3}$ in solving the *k*-mCAA problem is:

(26) $$Pr\left[\neg E_{3}\wedge \neg E_{4}\right]=Pr\left[\neg E_{3}\right]Pr\left[\neg E_{4}|\neg E_{3}\right]$$

Let gfrk be the probability at which $F_{B_{3}}$ is successful. As event $E_{4}$ occurs when $F_{B_{3}}$ fails:

(27) $$Pr[\neg E_{3}]=gfrk$$

Based on the general forking lemma [30,44] for $\gamma =q_{H_{3}}$ and |H|=p:

(28) $$\begin{array}{c} gfrk\geq acc_{3}\left(\frac{acc_{3}}{\gamma }-\frac{1}{p}\right)\geq {\left(1-\zeta \right)}^{\mu }\varepsilon \left(\frac{{\left(1-\zeta \right)}^{\mu }\varepsilon }{\gamma }-\frac{1}{p}\right) \end{array}$$

The probability at which the event $E_{4}$ does not occur, when the event $E_{3}$ does not occur, is equal to the probability at which the coin’s value of $\hat{\beta }$ valid forgeries is not equal to 0. Hence:

(29) $$Pr\left[\neg E_{4}|\neg E_{3}\right]=\zeta^{2}$$

Finally, the derivative $R_{3}$ of a successful solution to the *k*-mCAA problem is computed as described below:

(30) $$\varepsilon_{k-mCAA}^{R_{3}}\geq \zeta^{2}{\left(1-\zeta \right)}^{\mu }\varepsilon \left(\frac{{\left(1-\zeta \right)}^{\mu }\varepsilon }{\gamma }-\frac{1}{p}\right)$$

When p>>1, the expression is maximised at ζ=1/μ. Therefore:

(31) $$\begin{array}{cc} \varepsilon_{k-mCAA}^{R_{3}} & \geq \zeta^{2}\frac{{\left(1-\zeta \right)}^{2\mu }\varepsilon^{2}}{\gamma }=\\ \; & =\frac{1}{{\left(\mu +1\right)}^{2}}{\left(1-\frac{1}{\mu +1}\right)}^{2\mu }\frac{\varepsilon^{2}}{\gamma }\geq \\ \; & \geq \frac{\varepsilon^{2}}{\gamma e{\left(\mu +1\right)}^{2}}=\frac{\varepsilon^{2}}{\gamma e{\left(\left(q_{I}+q_{E}+q_{T}+q_{S}\right)+1\right)}^{2}} \end{array}$$

□

## 5. Performance Analysis

We compared our proposed IE-RCBS-kCAA scheme in terms of performance to two other schemes of similar design. For computational comparisons, we use notations of time-consuming operations based on results presented in Y. Huang et al. [39]:

- $T_{p}$: the time of executing a bilinear pairing operation $\hat{e}:$$G_{1}\times G_{2}\to G_{T}$;
- $T_{m}$: the time of executing a scalar multiplication in $G_{1}$ and $G_{2}$;
- $T_{e}$: the time of an exponentiation operation in $G_{T}$;
- $T_{h}$: the time of executing a map-to-point hash function.

Many operations are several orders of magnitude faster than bilinear pairing ($T_{p}$), map-to-point hash ($T_{h}$), scalar multiplication ($T_{m}$) in $G_{1}$ or $G_{2}$ and exponentiation in $G_{T}$ ($T_{e}$). These operations are hashing, inversion in $Z_{p}^{*}$, multiplication in $Z_{p}^{*}$, addition in $Z_{p}^{*}$, multiplication in $G_{T}$, and addition with $G_{1}$ or $G_{2}$. Therefore, we only consider $T_{p}$, $T_{m}$, $T_{e}$ and $T_{h}$ when we compare the computation costs of analysed signature schemes.

Table 2 includes a comparison of the IE-RCBS-kCAA scheme to two other schemes ($|G_{1}|$, $|G_{2}|$ and $|Z_{p}|$ mean the bit lengths of the elements in $G_{1}$, $G_{2}$ and $Z_{p}$, respectively). Our scheme has the same level of security as the two other schemes. Furthermore, our scheme IE-RCBS-kCAA contains a comparable number of time-consuming operations. In comparison with other schemes, the most time-consuming algorithm in IE-RCBS-kCAA is the *Sign* algorithm. However, the signature verification *Verify* algorithm is less time-consuming and requires only two bilinear pairing calculations instead of four. This feature of our algorithm is significant because, in practice, the signature operation is performed once, and the verification operation can be performed many times. However, obtaining this property resulted in our scheme’s larger signature size.

The run times of the *Sign* and *Verify* algorithms were examined using a test computer (Intel Core i7-8750H@2,20 GHz, 16 GB RAM) with a single thread. The scheme was implemented using an IAIK ECCelerate library for Java. Type 2 asymmetric pairing (ate pairing) based on the Barreto–Naehrig curve was used with different field sizes (160, 256, 384, 512 and 638 bits). The results (Table 3) average five repetitions. The time required to execute the *Sign* and *Verify* algorithms is less than 100 ms (except for the filed size of 638 bits). In Table 3, we also give the size in bytes of the signature for the different field sizes. We obtained this value by serialising all signature elements into an array of bytes (the signature consists of one element in $G_{2}$ and 3 in $Z_{p}$). The resulting signature size ranges from 387 to 1250 bytes. In real applications, adding a few dozen more bytes for formatting and metadata would be necessary.

## 6. Conclusions

In the IE-RCBS-kCAA scheme, a trusted status authority (TSA) directly performs the revocation procedure and, as a result, alleviates the load of the trusted authority (TA). Moreover, the architecture framework proposed in this paper can contain many TSA authorities, effectively solving the scalability problem. This property is unique and is not available in other similar solutions. Unlike other proposals, e.g., Jia et al. [27], obtaining such a solution is possible because a master private status key can be generated independently by each TSA.

We also formalize the security model for an adversary that can obtain a valid signature in the super sign query phase relevant to the public key selected by itself without providing the corresponding secret key. This security model proves that our IE-RCBS-kCAA signature scheme is semantically secure against Super Type I/II/III attackers under the *k*-mCAA and the DL assumptions over the respective groups $G_{2}$ and $G_{1}$.

The digital signature schemes must include a revocation mechanism to support non-repudiation and achieve Girault trust level 3. In order to achieve such properties, the paper proposes an architecture framework adapted to the needs of the IE-RCBS-kCAA scheme. In this scheme, an important role from the point of view of practical applications is played by the trusted status authority (TSA, see Figure 1), which periodically updates the explicit short-term certificates as long as the long-term explicit certificate has not been revoked. It is worth noting that an architecture framework (see Figure 1) can contain many TSA authorities, which effectively solves the scalability problem. This feature is very important when the TSA is applied to updating the validity of many short-term explicit certificates in the same or different trust domains.

The performance comparisons with two other revocable signature schemes show that our signature scheme has a similar number of time-intensive operations. The empirical execution times of the *Sign* and *Verify* algorithms are significantly below one second. The signature size in our scheme is approximately 1 KB. Such a size will be too large a value for some practical applications, e.g., the transmission of short messages from IoT sensors. However, this value will be within an acceptable range for large documents and for signing transactions that are sent over high-speed internet connections. The performance analysis shows that the scheme is suitable for further practical work when signing large documents is needed. In this case, the signature is always decidedly shorter than the message. In other practical applications, one must carefully analyse if the signature size is acceptable.

Future research includes deploying the signature scheme with the certificates to be validated according to the chain model (see Section 1). Such a signature scheme extends the feature of the IE-RCBS-kCAA scheme and should provide a non-repudiation property over a long period. Another important research problem will concern potential inconsistencies between the proposed mathematical proof of security of the encryption scheme and its real implementation. To compare different security models and evaluate their impact on the encrypted scheme, we plan to use a ranking based on pairwise comparisons (first proposed in Janicki et al. [45]). This approach will allow us to locate inconsistencies in the adopted security models and improve the design process of the encryption scheme.

## Figures and Tables

**Table 1:** The adversary types with different capabilities.

Adversary Type	TA Master Key	TSA Master Key	Implicit Certificate	Short-Term Explicit Certificate	User’s Secret Key	Public Key Replacement
$A_{1}$ (*non-certified user*)	no	no	no	yes	yes	yes
$A_{2}$ (*certified user*)	yes	yes	yes	yes	no	no
$A_{3}$ (*user with revoked certificate*)	no	no	yes	no	yes	yes
$A_{4}$ (*certified user*)	yes	no	yes	yes	no	no
$A_{5}$ (*non-certified user*)	no	yes	no	yes	yes	yes

**Table 2:** Comparisons between our scheme and two other schemes.

Scheme	Type	Signature Size	Sign	Verify	Security Level
SZS Y. Sun et al. [24]	CLS	$2|G_{1}|$	$3T_{m}+2T_{h}$	$4T_{p}+3T_{h}$	Super for $A_{1}$, $A_{2}$, $A_{3}$
HTH Y. Huang et al. [39]	CLS	$|G_{1}|$	$2T_{m}+2T_{h}$	$4T_{p}+T_{m}+3T_{h}$	Super for $A_{1}$, $A_{2}$, $A_{3}$
Proposed IE-RCBS-kCAA	IE-CBS	$|G_{2}\left|+3\right|Z_{p}|$	$T_{p}+5T_{m}$	$2T_{p}+4T_{m}$	Super for $A_{1}$, $A_{2}$, $A_{3}$

**Table 3:** Sign and Verify execution time, signature size.

Field Size (bits)	Sign (ms)	Verify (ms)	Signature Size (bytes)
160	20	12	387
256	28	23	560
384	50	45	789
512	79	70	1020
638	138	175	1250

## Data Availability

Data is contained within the article.
